# Comparative Error Analysis of GPT-5.2 Reasoning Modes on Performance in Postgraduate Internal Medicine Single-Best-Answer Questions

**DOI:** 10.7759/cureus.114680

**Published:** 2026-08-17

**Authors:** Hiruna K Diyasena, Aadam Ahmad, Thenuk H Weerasinghe, Olivia V Crawshaw, Anna A O'Brien

**Affiliations:** 1 General Internal Medicine, Royal Free London NHS Foundation Trust, London, GBR; 2 Orthopedics and Traumatology, Buckinghamshire Healthcare NHS Trust, Aylesbury, GBR; 3 General Internal Medicine, University College London, London, GBR; 4 Pediatrics, Royal Free London NHS Foundation Trust, London, GBR

**Keywords:** clinical reasoning, cognitive bias, large language models, medical education, postgraduate medical assessment

## Abstract

Introduction

Large language models (LLMs), such as Generative Pre-trained Transformer (GPT)-5.2 (OpenAI, Inc., San Francisco, United States), are increasingly used as revision tools for postgraduate medical examinations, including the Membership of the Royal College of Physicians (MRCP) Part 1 assessment. The educational value of newer extended-reasoning modes within the same model remains largely unexplored in the literature, and it is unclear whether improvements in performance or changes in the nature and frequency of errors can justify their greater computational cost.

Materials and methods

A paired design was used on a sample paper comprising 193 MRCP Part 1 single-best-answer (SBA) questions, with each question submitted three times to each mode: low-latency GPT-5.2 Instant and deeper-reasoning GPT-5.2 Thinking. The primary analysis compared the two modes at the question level. For each mode, a question was classified as majority correct when at least two of its three trials were correct, and the paired majority outcomes were analyzed using McNemar’s test. A secondary trial-level analysis used a logistic generalized estimating equation (GEE) model to account for repeated observations within questions. Incorrect responses were isolated and independently categorized by two reviewers blinded to mode using an adapted taxonomy of cognitive bias-like errors. Owing to sparse error counts, the findings were analyzed descriptively.

Results

Instant answered 549/579 correctly (94.8%), and Thinking answered 563/579 correctly (97.2%). Out of 193 questions, both modes achieved majority-correct responses in 183 questions (94.8%) and majority-incorrect responses in three questions (1.6%). There was a total of seven discordant pairs. Discordant-pair analysis found no statistically significant difference between modes (odds ratio (OR) = 6.0; 95% CI (0.72 - 49.84); P = 0.125), whereas trial-level analysis adjusted for operating mode alone showed higher odds of a correct response in favor of Thinking mode (OR = 1.92, 95% CI (1.14 - 3.24), P = 0.014). Instant mode errors were concentrated in input-conflicting hallucination and availability bias, whereas Thinking mode errors were more evenly distributed and included a greater relative proportion of reasoning hallucination.

Conclusion

Both GPT-5.2 reasoning modes achieved high and comparable performance in MRCP Part 1 SBA questions. Thinking mode was marginally more consistent across trials, but ceiling effects limited any practical benefit. Hence, low-latency modes may offer comparable practical utility in this structured, recall-dominant assessment context. Thinking mode demonstrated different error patterns when compared to Instant mode, but did not eliminate errors entirely; learners should critically appraise all model outputs.

## Introduction

Large language models (LLMs) are increasingly used in medical education, research, and assessment preparation [1]. The latest iterations of several LLMs, such as Generative Pre-trained Transformer (GPT)-5.2 (OpenAI, Inc., San Francisco, United States), now offer selectable operating modes that differ in latency and reasoning effort. This is achieved through variable utilization of tokens, which are small text units that the model processes and can ‘spend’ internally on deliberation. ‘Instant’ modes use fewer reasoning tokens, offering a lower-latency response with limited exploration of alternative explanations or self-checking. Conversely, ‘Thinking’ modes allocate more tokens to intermediate steps, enabling deeper reasoning and supporting complex multi-step tasks such as planning, debugging, and logical problem-solving [2]. The capacity of modern LLMs to answer complex medical problems, while allowing users to select reasoning mode based on task requirements, has contributed to their rapid adoption by medical trainees, particularly for question-based learning during examination preparation. As use of artificial intelligence (AI) increases, there is an ongoing need for rigorous analysis of LLM performance against current postgraduate medical assessments. The Membership of the Royal College of Physicians (MRCP) Part 1 is one such high-stakes postgraduate assessment required for progression within internal medicine specialties in the United Kingdom [3]. It consists of single-best-answer (SBA) multiple-choice questioning that requires factual recall, interpretation of clinical vignettes, and application of guidelines. 

Prior studies have shown that LLMs can achieve high accuracy on MRCP-style questioning [4,5]. However, most published literature evaluates LLMs as single systems, without accounting for differences in operating modes. While extended reasoning modes have demonstrated benefit in complex multi-step tasks [6], it remains unclear whether these incremental benefits translate to recall-dominant assessment formats such as SBA examinations. Answer accuracy alone may be insufficient for evaluating educational value since learners rely on accompanying answer explanations. LLM-generated explanations can contain hallucinated facts, unsupported inferences, or flawed reasoning, which may be particularly relevant during revision since plausible but incorrect explanations can be internalized by learners as misconceptions [1]. Whether the frequency or nature of such reasoning errors differs between low-latency and extended-reasoning modes within the same LLM remains poorly understood. This has important implications for learners because recognizing recurrent error patterns may support more critical appraisal of the validity and accuracy of LLM-generated explanations. 

Furthermore, these systems are constantly evolving, and up-to-date analyses of new model versions and respective operating modes are imperative in characterizing their uses, reliability, and limitations in a real-world context. As LLMs move from individual use to wider deployment as AI learning tools in educational platforms, their cumulative environmental burden becomes increasingly significant. Recent evidence involving medical applications has shown that more resource-intensive LLMs can consume substantially more energy and contribute more to carbon footprints without proportional gains in accuracy [7]. This supports the idea that resource-intensive modes are most defensible when they provide a demonstrable benefit over low-latency alternatives; however, there is limited supporting evidence for this in a medical examination context. Establishing whether reasoning modes improve exam performance in recall-dominant assessment tasks is therefore not only relevant to medical education but also more broadly to the governance of AI use.

Objectives

The primary objective was to quantitatively compare the performance of GPT-5.2 Instant and GPT-5.2 Thinking modes on MRCP Part 1-style SBA questions using question-level majority correctness and trial-level accuracy. The secondary objective was to qualitatively compare the frequency and distribution of cognitive bias-like and reasoning-error categories among incorrect responses. To our knowledge, this is an area that remains relatively unexplored in the literature.

## Materials and methods

Study design

A quantitative analysis using a paired design assessed two LLM operating modes, GPT-5.2 Instant and GPT-5.2 Thinking, on the same MRCP Part 1 sample paper. A further qualitative analysis of incorrect responses was performed based on an adapted taxonomy of cognitive biases and reasoning errors. 

Setting and data measurement

An official sample paper of 193 MRCP Part 1 SBA questions was used. Predefined answer keys and rationales were provided by the examination board. Questions spanned multiple core specialties and clinical competencies, which were categorized according to the official MRCP Part 1 content map by two independent reviewers (AA and HKD). Discrepancies were resolved by consensus agreement; no third-party adjudication was required.

The study was conducted through OpenAI’s ChatGPT-5.2 (December 2025) web interface using the user-visible model labels GPT-5.2 Instant and GPT-5.2 Thinking. All evaluations were performed using the ChatGPT Plus subscription tier, which allowed unlimited access to the GPT-5.2 Instant and GPT-5.2 Thinking modes. No immutable model snapshot or interface build identifier was displayed or accessible during data collection. The study was designed to evaluate the established GPT-5.2 operating modes as experienced by medical learners. The preset internal configurations for GPT-5.2 Instant and Thinking were not visible or accessible to users. All other user-facing settings were unmodified, and all evaluations were performed using the default configuration available through the interface during the study period. The complete underlying inference configuration of these operating modes, including sampling temperature, random seed, top-p, system instructions, decoding strategy, and reasoning-token allocation, was proprietary to the platform and was not disclosed by the model designers. Consequently, these parameters could not be independently verified, fully standardized, or reported.

All evaluations were performed without time constraints, external tools, feedback, or examples, using a zero-shot prompting approach. Remote browser data access was disabled. Browser history and cache were cleared between each data collection session. To minimize memory bias, each prompt was entered into the ‘New chat’ function. Each prompt consisted solely of the unaltered question vignette, lead-in, and five answer options. No additional contextual inputs or guidance were provided. A definitive response was defined as the selection of a single answer option only. If an output contained multiple answer selections, no definitive answer, or an equivocal conclusion, one standardized clarification prompt would have been provided: “Please select the single-best-answer from the options provided.” An output that remained non-definitive after this clarification would have been classified as an incorrect response. If an output produced a single answer selection without an accompanying explanation, including after clarification, one standardized prompt would have been provided: "Please explain the reasoning for your selected answer." The original answer selection would have remained the response used for quantitative accuracy analysis, while the subsequent explanation would have been retained only for qualitative error classification if the answer was incorrect. The standardized input and response-handling workflow is shown in Appendix 1. Each question was submitted manually into GPT-5.2 in the order presented in the official sample paper, without randomization. Each question was submitted three separate times into each mode as independent trials across different days. This three-trial approach was informed by previous research in which medical examination questions were submitted three times and aggregate performance remained broadly consistent across the three rounds [8]. Answers were collected between December 16, 2025, and February 1, 2026. 

All GPT-5.2 answer outputs were recorded in Microsoft Excel (Microsoft Corporation, Redmond, Washington, United States) and subsequently labeled as correct or incorrect based on whether the GPT-5.2 output matched the predefined answer key. Correct answer selection by either option label or unambiguous option text was accepted. All incorrect responses were collated for further qualitative analysis. Non-definitive incorrect responses were classified using the stated reasoning if provided. Where no reasoning was provided, the response was recorded as unclassifiable for qualitative analysis. Two independent reviewers (AAO and HKD) identified the primary reasoning error for incorrect responses by comparing each incorrect response with the official answer rationale and identifying the point at which the model's answer explanation first diverged from the correct clinical reasoning pathway. Reviewers then classified this divergence using a predetermined taxonomy adapted from established literature (Table 1) [9,10]. The taxonomy included commonly reported cognitive biases and reasoning errors encountered during clinical tasks by LLMs, together with definitions and illustrative case examples to support classification. Where more than one error category was potentially applicable, reviewers recorded the category that best represented the principal error directly responsible for the incorrect answer explanation. Errors stemming from that primary reasoning failure were considered downstream errors and were not coded separately. Only one primary error category was therefore assigned to each incorrect response. To reduce classification bias, reviewers were blinded to the reasoning mode when categorizing incorrect responses. Discrepancies were resolved by consensus agreement; no third-party adjudication was required. 

**Table 1:** A taxonomy of biases in large language model outputs during medical reasoning tasks Taxonomy of cognitive biases and reasoning errors produced by large language models during medical reasoning tasks, adapted from Saposnik et al. [9] and Kim et al. [10], with definitions and illustrative case examples. ECG: electrocardiogram; LLM: large language model; PET: positron emission tomography

Type of Bias	Definition	Example
Anchoring Bias	Over-reliance on initial clinical information (the “anchor”) despite later contradictory information.	“Patient X has a known history of epilepsy and presents after a collapse. Their ECG shows complete heart block.” The LLM selects seizure due to anchoring on the past medical history while ignoring later evidence supporting cardiac syncope.
Availability Bias	Overestimating a diagnosis because it is familiar or likely over-represented in the LLM training data, leading to neglect of underlying base rates.	“Patient X is a young woman with a mobile breast lump.” The LLM selects breast cancer as the most likely diagnosis, which is salient but neglects base rates, as this is far less common in this patient demographic than a fibroadenoma.
Confirmation Bias	Seeking and favoring specific information that supports a pre-existing diagnosis while ignoring contradictory evidence.	“Patient X has epigastric pain radiating into the left arm associated with vomiting.” The LLM selects gastritis by emphasizing the abdominal pain and vomiting while discounting features supporting myocardial infarction, such as radiation of pain into the arm.
Framing Effect	The tendency for clinical decisions to be influenced by the way information is presented rather than by its objective content.	“Patient X is offered a treatment with a 90% survival rate.” The LLM selects this option but rejects the identical treatment when framed as having a 10% mortality rate.
Premature Closure	Accepting a plausible diagnosis before all relevant evidence has been considered.	“Patient X has fatigue and microcytic anemia. They report a five-month history of weight loss and altered bowel habit.” The LLM selects iron deficiency anemia after the opening sentence and stops before identifying colorectal cancer as a plausible option.
Omission Bias	Failing to acknowledge, evaluate, or act on a key clinical detail present in the stem.	“Patient X has atrial fibrillation and active gastrointestinal bleeding.” The LLM selects to treat with anticoagulation while failing to recognize the clinically significant bleeding risk.
Commission Bias	Adding an unnecessary diagnosis, investigation or treatment beyond what available evidence supports.	“Patient X has an uncomplicated upper respiratory tract infection with normal observations.” The LLM selects to treat with antibiotics despite no evidence to support this action.
Overconfidence	Expressing certainty that is disproportionate to the available clinical evidence.	“Patient X has non-specific abdominal pain and weight loss without definitive investigation results.” The LLM states that pancreatic cancer is the most likely diagnosis despite insufficient evidence to establish the diagnosis.
Representativeness Bias	Choosing a diagnosis because the case resembles a textbook pattern, leading to errors when atypical presentations are encountered.	“Patient X is elderly and presents with global memory impairment, loss of appetite and social withdrawal but denies low mood.” The LLM selects dementia because the presentation does not resemble textbook depression, overlooking that depression may present atypically in older adults.
Satisficing Bias	The tendency to accept the first option that meets a minimum threshold of plausibility without searching for the optimal answer.	“Patient X has severe hyperkalemia with ECG changes.” The LLM selects nebulized salbutamol as the preferred initial treatment option because it lowers potassium but fails to continue evaluating the options and identify intravenous calcium as the most urgent treatment.
Confabulation / Non-factual Hallucination	Generating plausible clinical information with no basis in the stem or medical knowledge which is entirely fabricated.	“Patient X has suspected polymyalgia rheumatica.” The LLM invents a non-existent diagnostic antibody test and uses its supposed result to justify the diagnosis.
Factual Hallucination	Generating information that directly contradicts established, verifiable medical knowledge.	“Patient X has severe asthma but also requires treatment for essential tremor.” The LLM selects propranolol while incorrectly claiming that it does not cause bronchospasm.
Input-Conflicting Hallucination	The model generates a response that contradicts, misinterprets, or misrepresents the information provided in the input.	“Patient X has a small cell lung cancer. Which investigation is most useful in assessing suitability for resection?” The LLM selects lung function tests as it misinterprets the question as assessing operative fitness rather than first determining oncological resectability through staging with a PET scan.
Reasoning Hallucination	The model produces a response in which the chain of reasoning is internally flawed or logically incomplete.	“Patient X has sudden-onset testicular pain with a negative Prehn’s sign.” The LLM correctly recognizes a high clinical suspicion of testicular torsion and the need for urgent surgical exploration, but incorrectly reasons that testicular ultrasonography should be performed first to confirm the diagnosis.
Memory-Based Hallucination	The model generates clinical recommendations that were accurate at the time of its training data but have since been superseded, withdrawn, or revised.	“Patient X has non-valvular atrial fibrillation and requires anticoagulation.” The LLM recommends warfarin from an outdated guideline rather than a direct oral anticoagulant as per more recent guidance.

Overall accuracy was defined as the proportion of correct trial-level responses in each operating mode. The primary paired comparison used majority correctness per question, defined as two or more correct responses across three trials within each mode. A secondary generalized estimating equation (GEE) analysis compared trial-level correctness while accounting for repeated observations within questions. Secondary outcomes included the descriptive distribution of cognitive bias and reasoning-error categories among incorrect responses.

Statistical methods

Descriptive statistics were used to analyze the data overall as well as by specialty and competency. The inter-reviewer agreement for categorization of question items by specialty and competency, as well as the cognitive bias category for incorrect responses, was calculated using Cohen’s kappa value. Given small sample sizes across specialty, competency, and bias categories, subgroup analyses should be interpreted as descriptive and were not powered for statistical inference. To assess whether the performance of Thinking mode differed from Instant mode, majority correctness per question was derived by aggregating three independent trials per mode, after which paired comparisons were performed using McNemar’s test. This test evaluated whether the number of discordant pairs favoring each mode differed significantly. Effect size was reported as a matched-pairs odds ratio, with the 95% confidence interval calculated on the log scale using the standard error derived from discordant counts. A secondary analysis using a GEE model accounted for repeated observations per question. Correctness (correct vs. incorrect) was modeled using a binomial distribution and a logit link function, with repeated observations grouped according to the examination question from which they were generated. An exchangeable working correlation structure and robust (sandwich) standard errors were used to account for within-question correlation. Type III Wald χ² statistics were used for hypothesis testing, and exponentiated regression coefficients were reported as odds ratios with 95% Wald confidence intervals. More complex GEE models involving combinations of trial number, specialty, and competency as additional predictors were explored but were unstable due to quasi-complete separation arising from sparse incorrect responses. A reduced model including operating mode alone was therefore fitted. A two-sided P-value < 0.05 was considered statistically significant. All analyses were conducted using IBM SPSS Statistics for Windows, Version 31 (Released 2025; IBM Corp., Armonk, New York, United States). Results were tabulated and graphed in Microsoft Excel Version 16.105.3.

Ethics statement

This study did not involve human participants, identifiable personal data, or animal subjects. Therefore, review and approval by the institutional review board were not required. The research consisted solely of secondary analysis of educational assessment material from “The Federation of the Royal Colleges of Physicians of the UK” [11]. Official permission to use the item bank materials and approval to publish were obtained from the copyright holders.

## Results

Overall performance

**Figure 1:**
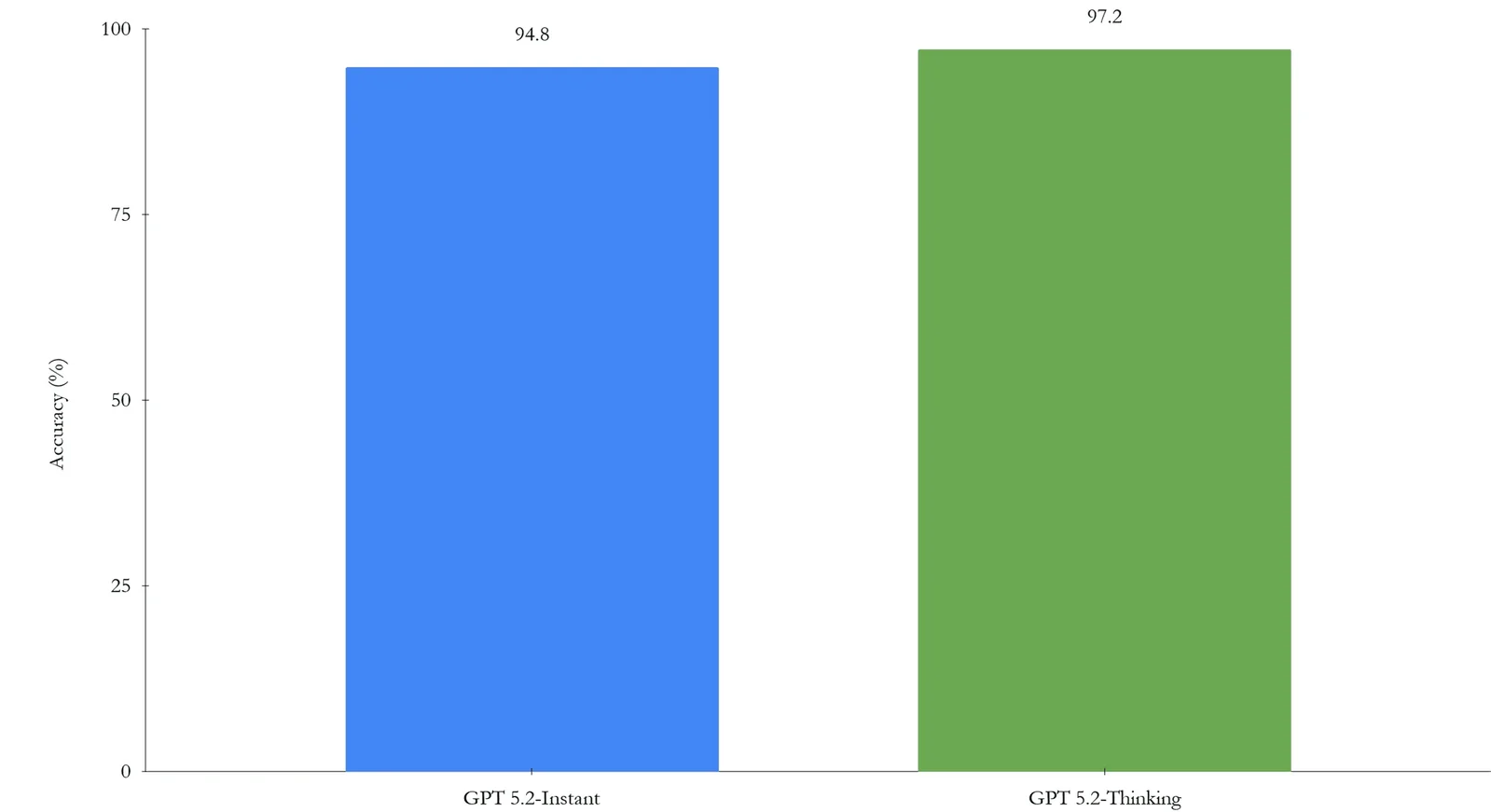
Overall accuracy of GPT-5.2 Instant and Thinking modes The figure shows overall accuracy across 579 responses per reasoning mode, GPT-5.2 Instant and GPT-5.2 Thinking, for MRCP Part 1 single-best-answer questions. GPT: Generative Pre-trained Transformer; MRCP: Membership of the Royal College of Physicians

Across all trials, all outputs contained a single definitive answer corresponding to one answer option with an accompanying answer explanation on the first attempt. No responses contained multiple answer selections or equivocal conclusions. No responses omitted a final answer or answer explanation. Hence, further clarification prompting was not required in our study. Out of 579 responses involving 193 MRCP Part 1 questions repeated across three trials, GPT-5.2 Instant answered 549 correctly (94.8% accuracy), and GPT-5.2 Thinking answered 563 correctly (97.2% accuracy). Figure 1 highlights the difference between the two modes, with GPT-5.2 Thinking scoring 2.4% higher overall. Out of 193 questions, both modes achieved majority-correct responses in 183 questions (94.8%) and majority-incorrect responses in three questions (1.6%). There were a total of seven discordant pairs. Thinking achieved majority-correct performance in six questions where Instant did not, compared with one question where Instant outperformed Thinking, corresponding to a matched-pairs odds ratio (OR) of 6.0 (95% CI 0.72 - 49.84). This difference did not reach statistical significance on McNemar testing (p = 0.125), reflecting the small number of discordant questions. A GEE model clustering repeated observations by question demonstrated higher odds of a correct response with the Thinking mode compared to the Instant mode (OR = 1.92, 95% CI (1.14 - 3.24), p = 0.014).

Performance by specialty

**Figure 2:**
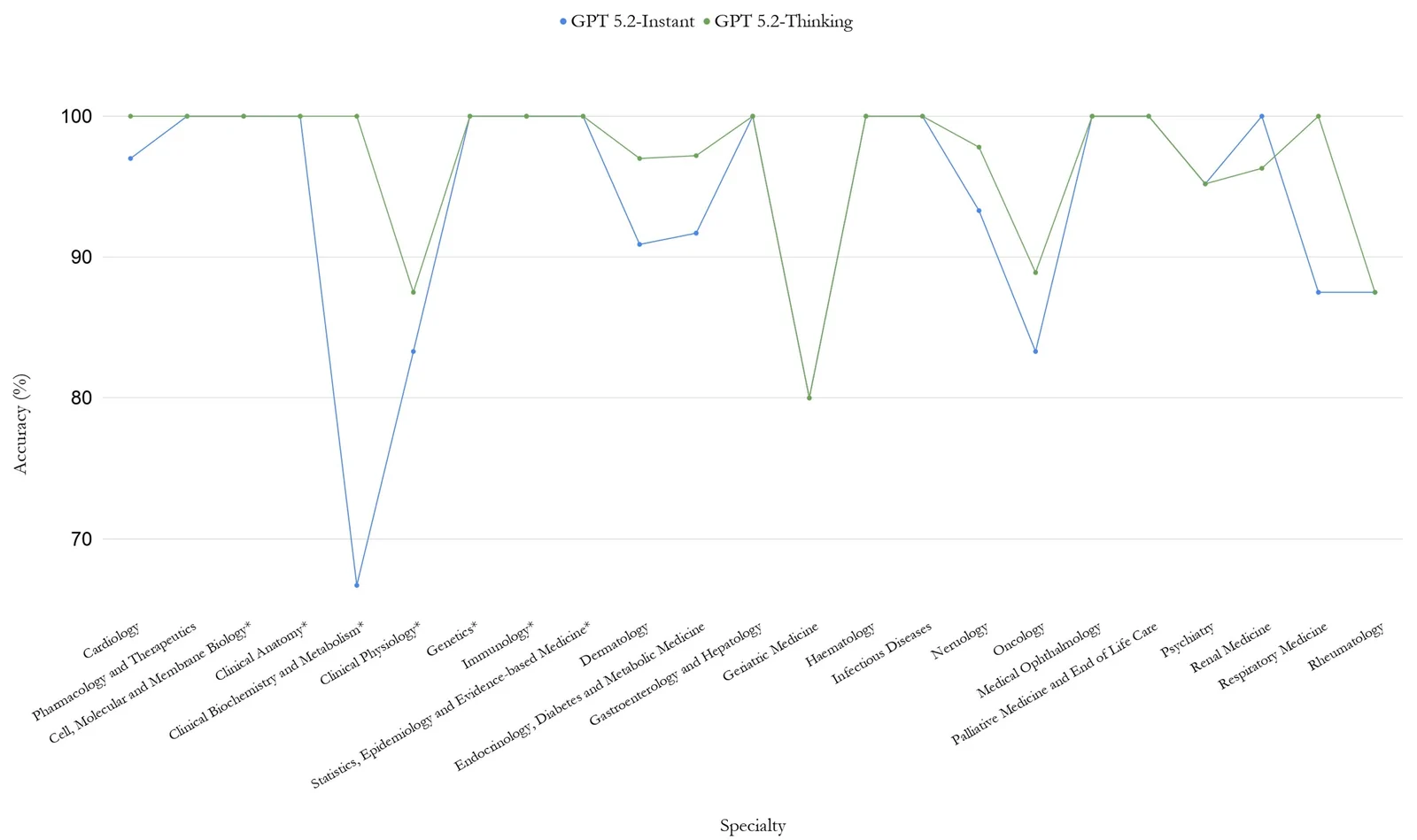
Performance comparison of GPT-5.2 Instant and Thinking modes in MRCP Part 1 questions by specialty The figure shows a graphical representation of accuracy across MRCP Part 1 specialty categories. Subspecialties within ‘Clinical Sciences’ are marked with an asterisk (*). GPT: Generative Pre-trained Transformer; MRCP: Membership of the Royal College of Physicians

Table 2 provides a breakdown of performance between the modes across medical specialties. Inter-rater agreement using Cohen’s kappa was near-perfect when categorizing medical specialty (κ = 0.978, SE = 0.011, p < 0.001). Overall, both modes delivered high accuracy throughout all specialties, with scores ranging from 66.7% to 100% for Instant and 80% to 100% for Thinking (Figure 2). Both Thinking and Instant scored equally in 14/23 specialties. Thinking mode scored higher than Instant mode in 8/23 specialties. Instant scored higher than Thinking in 1/23 specialties. The largest specialty in the exam by proportion of questions was ‘Clinical Sciences’ with 19.2%.

**Table 2:** Performance comparison of GPT-5.2 Instant and Thinking modes in MRCP Part 1 questions by specialty The table presents trial-level accuracy across MRCP Part 1 specialty categories. Difference represents Thinking mode accuracy minus Instant mode accuracy. GPT: Generative Pre-trained Transformer; MRCP: Membership of the Royal College of Physicians

Specialty	Number of Responses (N)	Instant Mode Accuracy, % (No./Total)	Thinking Mode Accuracy, % (No./Total)	Difference (+/- %)	% of Total N
Cardiology	33	97.0 (32/33)	100.0 (33/33)	+3.0	5.7
Clinical Pharmacology and Therapeutics	57	100.0 (57/57)	100.0 (57/57)	0.0	9.8
Clinical Sciences - Cell, Molecular and Membrane Biology	12	100.0 (12/12)	100.0 (12/12)	0.0	2.1
Clinical Sciences - Clinical Anatomy	12	100.0 (12/12)	100.0 (12/12)	0.0	2.1
Clinical Sciences - Clinical Biochemistry and Metabolism	9	66.7 (6/9)	100.0 (9/9)	+33.3	1.6
Clinical Sciences - Clinical Physiology	24	83.3 (20/24)	87.5 (21/24)	+4.2	4.1
Clinical Sciences - Genetics	18	100.0 (18/18)	100.0 (18/18)	0.0	3.1
Clinical Sciences - Immunology	18	100.0 (18/18)	100.0 (18/18)	0.0	3.1
Clinical Sciences - Statistics, Epidemiology and Evidence-based Medicine	18	100.0 (18/18)	100.0 (18/18)	0.0	3.1
Dermatology	33	90.9 (30/33)	97.0 (32/33)	+6.1	5.7
Endocrinology, Diabetes and Metabolic Medicine	36	91.7 (33/36)	97.2 (35/36)	+5.5	6.2
Gastroenterology and Hepatology	36	100.0 (36/36)	100.0 (36/36)	0.0	6.2
Geriatric Medicine	15	80.0 (12/15)	80.0 (12/15)	0.0	2.6
Hematology	27	100.0 (27/27)	100.0 (27/27)	0.0	4.7
Infectious Diseases	48	100.0 (48/48)	100.0 (48/48)	0.0	8.3
Neurology	45	93.3 (42/45)	97.8 (44/45)	+4.5	7.8
Oncology	18	83.3 (15/18)	88.9 (16/18)	+5.6	3.1
Medical Ophthalmology	21	100.0 (21/21)	100.0 (21/21)	0.0	3.6
Palliative Medicine and End of Life Care	3	100.0 (3/3)	100.0 (3/3)	0.0	0.5
Psychiatry	21	95.2 (20/21)	95.2 (20/21)	0.0	3.6
Renal Medicine	27	100.0 (27/27)	96.3 (26/27)	-3.7	4.7
Respiratory Medicine	24	87.5 (21/24)	100.0 (24/24)	+12.5	4.1
Rheumatology	24	87.5 (21/24)	87.5 (21/24)	0.0	4.1
Total	579	94.8	97.2	+2.4	100.0

Performance by competency

**Figure 3:**
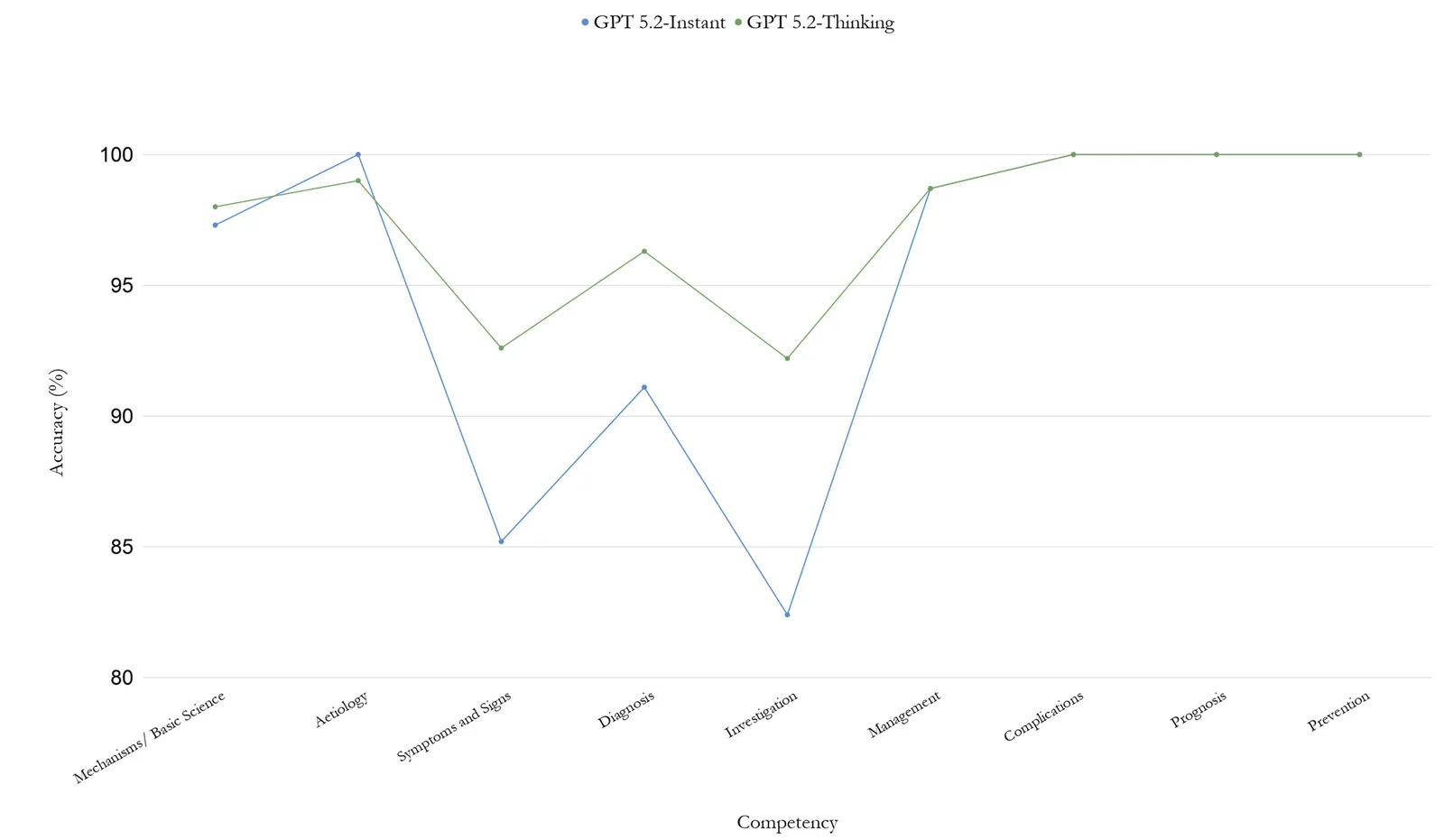
Performance comparison of GPT-5.2 Instant and Thinking modes in MRCP Part 1 questions by competency The figure shows a graphical representation of accuracy across MRCP Part 1 clinical competency domains. GPT: Generative Pre-trained Transformer; MRCP: Membership of the Royal College of Physicians

Table 3 provides a breakdown of performance between the modes across medical competencies. Cohen’s kappa indicated high agreement between raters for the categorization of competencies (κ = 0.968, SE = 0.014, p < 0.001). Both modes delivered high performance throughout all clinical competencies, with scores ranging from 82.4% to 100% for Instant and 92.2% to 100% for Thinking (Figure 3). Both Thinking and Instant scored equally in 4/9 clinical competencies. Thinking outperformed Instant in 4/9 of the competencies, while Instant outperformed Thinking in 1/9. ‘Mechanisms/Basic Science’ accounted for the largest proportion of questions by competency, representing 25.9% of the total questions.

**Table 3:** Performance comparison of GPT-5.2 Instant and Thinking modes in MRCP Part 1 questions by competency The table presents trial-level accuracy across MRCP Part 1 clinical competency domains. Difference represents Thinking mode accuracy minus Instant mode accuracy. GPT: Generative Pre-trained Transformer; MRCP: Membership of the Royal College of Physicians

Competency	Number of Responses (N)	Instant Mode Accuracy, % (No./Total)	Thinking Mode Accuracy, % (No./Total)	Difference (+/- %)	% of Total N
Mechanisms/ Basic Science	150	97.3 (146/150)	98.0 (147/150)	+0.7	25.9
Etiology	102	100.0 (102/102)	99.0 (101/102)	-1.0	17.6
Symptoms and Signs	27	85.2 (23/27)	92.6 (25/27)	+7.4	4.7
Diagnosis	135	91.1 (123/135)	96.3 (130/135)	+5.2	23.3
Investigation	51	82.4 (42/51)	92.2 (47/51)	+9.8	8.8
Management	75	98.7 (74/75)	98.7 (74/75)	0.0	13.0
Complications	18	100.0 (18/18)	100.0 (18/18)	0.0	3.1
Prognosis	12	100.0 (12/12)	100.0 (12/12)	0.0	2.1
Prevention	9	100.0 (9/9)	100.0 (9/9)	0.0	1.6
Total	579	94.8	97.2	+2.4	100.0

Error analysis 

Incorrect responses were categorized using the predefined list of biases outlined in Table 1. Cohen’s kappa indicated high inter-reviewer agreement for categorization of errors into respective bias categories (κ = 0.782, SE = 0.066, p < 0.001). At the trial level (Table 4), where each incorrect model output was counted separately, 46 errors were identified, comprising 30 from GPT-5.2 Instant and 16 from GPT-5.2 Thinking. Instant errors were most commonly input-conflicting hallucinations (9/30, 30.0%) and availability bias (7/30, 23.3%). Thinking errors were most commonly availability bias (4/16, 25.0%), followed by input-conflicting hallucinations and reasoning hallucination (each 3/16, 18.8%). At the question level (Table 4), where repeated incorrect trials of the same question were collapsed within each mode, errors occurred across 12 Instant questions and 11 Thinking questions. Instant question-level errors most commonly involved input-conflicting hallucination and availability bias (each 3/12, 25%), while Thinking errors were more evenly distributed across availability bias, commission bias, input-conflicting hallucination, and reasoning hallucination (each 2/11, 18.2%). For each bias category, the reasoning mode with the higher proportion of errors was generally consistent at both the trial and question level. The only exceptions were availability bias, which was proportionally higher in Thinking at the trial level but higher in Instant at the question level, and commission bias, which showed the opposite pattern. Owing to the small number of incorrect responses and repeated observations within questions, these findings were analyzed descriptively without inferential comparison.

**Table 4:** Distribution of cognitive biases and reasoning errors by GPT-5.2 Reasoning mode for MRCP Part 1 questions Proportion of cognitive biases in GPT-5.2 Instant and GPT-5.2 Thinking at the trial-level and question-level. Bias categories from the predefined taxonomy with no observed examples in either mode are not shown in the results table. Percentage differences are descriptive only. GPT: Generative Pre-trained Transformer; MRCP: Membership of the Royal College of Physicians

Bias	Trial Level			Question Level		
Cognitive Bias	Instant n = 30 (% total)	Thinking n = 16 (% total)	Difference (%)	Instant n = 12 (% total)	Thinking n = 11 (% total)	Difference (%)
Input-Conflicting Hallucination	9 (30.0%)	3 (18.8%)	11.2%, Instant	3 (25.0%)	2 (18.2%)	6.8%, Instant
Availability Bias	7 (23.3%)	4 (25.0%)	1.7%, Thinking	3 (25.0%)	2 (18.2%)	6.8%, Instant
Commission Bias	4 (13.3%)	2 (12.5%)	0.8%, Instant	2 (16.7%)	2 (18.2%)	1.5%, Thinking
Confirmation Bias	3 (10.0%)	2 (12.5%)	2.5%, Thinking	1 (8.3%)	1 (9.1%)	0.8%, Thinking
Omission Bias	3 (10.0%)	0 (0.0%)	10.0%, Instant	1 (8.3%)	0 (0.0%)	8.3%, Instant
Premature Closure	3 (10.0%)	0 (0.0%)	10.0%, Instant	1 (8.3%)	0 (0.0%)	8.3%, Instant
Reasoning Hallucination	1 (3.3%)	3 (18.8%)	15.5%, Thinking	1 (8.3%)	2 (18.2%)	9.9%, Thinking
Anchoring Bias	0 (0.0%)	1 (6.3%)	6.3%, Thinking	0 (0.0%)	1 (9.1%)	9.1%, Thinking
Satisficing Bias	0 (0.0%)	1 (6.3%)	6.3%, Thinking	0 (0.0%)	1 (9.1%)	9.1%, Thinking
Total	30 (100%)	16 (100%)		12 (100%)	11 (100%)	

## Discussion

Key results

Of 579 responses, GPT-5.2 Instant scored 94.8%, and GPT-5.2 Thinking scored 97.2%, both well above the historical pass mark of approximately 62% [5]. There was high concordance between modes, with only seven discordant pairs. There was no statistically significant difference between reasoning modes in discordant-pair analysis (OR = 6.0, 95% CI (0.72 - 49.84), P = 0.125). However, trial-level analysis adjusted for mode alone identified significantly higher odds of a correct response with Thinking mode (OR = 1.92, 95% CI (1.14 - 3.24), p = 0.014). The greatest proportions of questions by specialty and competency were ‘Clinical Sciences’ (19.2%) and ‘Mechanisms/Basic Science’ (25.9%), respectively. Across both modes, errors clustered mainly around input misinterpretation, base-rate neglect, and flawed reasoning, which suggests recurrent patterns rather than random mistakes. Although Thinking produced fewer errors overall, its residual errors were more evenly distributed across bias categories, while Instant errors were more concentrated in input-conflicting hallucination and availability bias.

Interpretation of quantitative analyses

Both GPT-5.2 Instant and GPT-5.2 Thinking modes achieved high overall accuracy, suggesting a ceiling effect in this SBA examination format. The quantitative findings should therefore be interpreted cautiously both in terms of statistical significance and practical educational benefit. McNemar’s test was applied after three trials per mode had been collapsed into a binary majority outcome for each question; thus, some trial-level information was inherently lost through aggregation. The resulting small number of discordant pairs limited the statistical power of this type of analysis. The GEE model, on the other hand, retained all trial-level data while accounting for clustering by question. It could therefore detect smaller differences in correct answer consistency that would have otherwise been lost following majority aggregation. For example, a question answered correctly in two out of three Instant trials and three out of three Thinking trials would be concordant and thus not contribute to the McNemar analysis but would still be detectable to the GEE model. The observed discrepancy between the primary and secondary analyses can co-exist because they evaluate different aspects of performance. Broadly speaking, the primary analysis evaluates whether disagreements between the modes favor one over the other, whereas the secondary analysis assesses the likelihood of a correct answer on any single trial. The significance of the GEE result should most reliably be interpreted as evidence that extended reasoning modes in this recall-dominant assessment context may be less prone to occasional trial-level errors; however, in educational terms, the practical gain is limited when both modes already answer the majority of questions correctly. Discordant outputs between attempts more generally reveal underlying model uncertainty, although we did not formally test repeated prompting as a learner intervention. As such, repeated low-latency prompting should not be considered as a validated substitute for extended reasoning modes. Instead, discordant repeated outputs should act as a signal for learners to verify the answer against reputable sources rather than relying on the LLM output alone. 

While Thinking mode may provide limited practical benefit in this assessment context, its higher odds of producing a correct trial-level response remain informative from a mechanistic perspective. Extended inference modes, such as GPT-5.2 Thinking, may utilize internal processes similar to ‘chain-of-thought (CoT) prompting’ to generate intermediate reasoning steps to produce responses [12]. This has led to expected improvements in complex reasoning tasks involving multi-step inference and logical problem-solving [12,13]. However, in our dataset, MRCP Part 1 SBA questions were more constrained than traditional open-ended clinical reasoning tasks because they provide fixed answer options and largely test factual, scientific, knowledge-based content. The marginal benefits that extended reasoning offers in this context may have been achieved by allocating greater computational effort to stem interpretation, distractor comparison, and internal checking before choosing the correct answer. 

The broader medical LLM literature suggests that CoT prompting does not provide uniform benefit across all medical tasks. Liévin et al. showed that LLMs can answer and reason about challenging medical questions, but performance varies according to dataset, prompting strategy, and task structure [14]. Similarly, Jeon and Kim found that more complex CoT reasoning did not significantly influence performance in medical question answering; rather, data characteristics and model architecture were more important determinants [15]. Although manual CoT prompting discussed in the literature is conceptually similar to the processes within extended reasoning modes, the two are not equivalent. Internal model deliberation or other proprietary differences between modes may have contributed to differences in performance. As such, our results suggest that extended reasoning modes may confer a small consistency benefit that is not always captured by conventional CoT studies.

Interpretation of qualitative error analysis

The error taxonomy used in this study was adapted from Saposnik et al. [9] and Kim et al. [10], incorporating both traditional clinical cognitive biases (anchoring, availability bias, confirmation bias, and premature closure) and LLM-specific hallucination categories (input-conflicting, factual, and reasoning). This reflects the wide variety of errors that LLMs are susceptible to and provides a practical reference framework to aid learners when appraising LLM outputs. Our study demonstrated distinct error profiles for GPT-5.2 Instant and GPT-5.2 Thinking, although both modes demonstrated input-conflicting hallucination, availability bias, commission bias, confirmation bias, and reasoning hallucination. These consistent types of error may be related to similar vulnerabilities stemming from the same question material and limitations common to the underlying model. Omission bias and premature closure were observed only in GPT-5.2 Instant, whereas anchoring and satisficing bias were observed only in GPT-5.2 Thinking. Each occurred in small numbers and may be a broader indication that our overall categorization is susceptible to random sample variation and misclassification, especially since LLM outputs often contained multiple biases and reviewers were tasked to identify only the primary bias. Additionally, the small number of total incorrect responses (n = 46) precluded any reliable statistical comparison; hence, analysis should be interpreted as descriptive and hypothesis-generating only.

In terms of differences between modes, GPT-5.2 Instant showed the largest relative proportion of input-conflicting hallucination errors, while GPT-5.2 Thinking showed a comparatively greater proportion of reasoning hallucination errors. One possible explanation for this is that low-latency modes may allow less opportunity to reconcile the vignette, lead-in, and answer options, thus increasing the risk of question misinterpretation. Conversely, extended reasoning may reduce surface-level misinterpretation but could be vulnerable to propagation of early errors within reasoning chains to produce internally justified yet flawed outputs. However, these mechanisms remain speculative since they cannot be established from the observed outputs alone, and the visible explanation may not reliably represent the model’s internal deliberation process. Previous work supports the idea that errors arise when LLMs rely too heavily on internal parametric knowledge without adequately prioritizing or integrating prompt information, resulting in context-discordant outputs [10]; this may constitute a similar decision-making process to low-latency modes. Similarly, CoT studies suggest that intermediate reasoning can aid in task performance but introduces vulnerability to stepwise logical drift [16]. One example of Thinking’s reasoning hallucination involved a question from the MRCP Part 1 sample paper requiring identification of the pharmacological mechanism underlying a medication-induced electrolyte disturbance. Instant answered the question correctly in two of three trials, selecting the direct and well-established mechanism associated with the medication. Thinking, however, answered the question incorrectly in two of three trials after identifying the correct mechanism as “too trivial” and exploring alternative pathways, deviating further from the correct solution. 

Several studies support the relevance of this bias-focused approach. Maitland et al. found that GPT-4 errors on the same MRCP sample paper were predominantly factual, omission, and context errors, the latter of which broadly corresponds to the input-conflicting hallucination category seen in our study [4]. Given the relatively fewer factual hallucinations seen in our study as well as a similar predominance for input-conflicting hallucination, incomplete use of key stem information seems to persist across model generations despite relatively fewer factual errors. Schmidgall et al. demonstrated in BiasMedQA that LLM performance can decline when questions contain bias-inducing features [17]. Wang and Redelmeier similarly showed that generative AI models can demonstrate human-like cognitive biases in medical recommendations, sometimes exceeding those observed in clinicians [18]. A subsequent study found that forewarning LLMs to consider cognitive biases increases response length and explicit reflection but only marginally reduces and does not eliminate overall bias [19]. The limited literature directly examining reasoning models is particularly pertinent. Taken together, these studies suggest that extended reasoning may reduce some forms of cognitive bias but does not abolish bias and may introduce different failure modes. As such, further research is needed to better characterize and understand methods to reduce bias in outputs.

Comparison with existing literature

There is limited research that compares GPT reasoning modes in a medical context. Proff et al. compared GPT-5 Instant and Thinking modes in the context of clinical guideline-based radiology questions rather than SBA examination questions, with correct response rates of 88.5% and 96.5%, respectively [20]. Their finding that Thinking mode outperformed Instant mode is directionally consistent with our trial-level analysis, which also demonstrated higher odds of a correct response with Thinking mode. However, the magnitude and practical relevance of our findings were smaller. In contrast, Nedos et al. found poor performance for GPT-5 Instant in emergency department triage, with low agreement against physician-assigned Emergency Severity Index scores, highlighting that differences between reasoning modes may be more pronounced in complex clinical judgment-based scenarios [21]. Guideline and triage-based questions often require conditional reasoning, sequencing, and application of multi-step decision rules. It is possible that the pattern-recognition basis of Instant mode may fail to apply the conditional logic required in the application of guidelines or clinical triage but remains sufficient in an SBA context. Conversely, Iwabu et al. reported that GPT-5.2 Instant achieved higher agreement for anesthetic physical status classification with cardiovascular anesthesiologists when compared to GPT-5.2 Thinking, producing exact matches in 75% and 68.8% of cases, respectively [22]. Similarly, Kopka and Feufel found that GPT-5 reasoning modes produced variable benefit in care-seeking advice based on model version and task framing [23]. These findings collectively support task-dependent utility based on operating mode instead of a generalizable superiority for increased reasoning across medical contexts. 

GPT’s ability to answer MRCP Part 1 questions has been explored before in 2024 [5], with both GPT-3.5 and GPT-4 scoring 66.4% and 84.8%, respectively. Our findings extend this literature by showing substantially higher performance, with GPT-5.2’s Instant and Thinking modes achieving 94.8% and 97.2%, respectively. These scores sit at the higher end of published GPT-5 literature on SBA assessment performance, with previous studies achieving accuracies ranging from 69.1% to 96.5% in a variety of specialties, including ophthalmology, endocrinology, surgery, and internal medicine [24-27]. Antaki et al.’s paper similarly adjusted reasoning effort within the same GPT-5 model and showed that higher reasoning effort modes outperformed lower-latency versions by a small magnitude; however, their findings were restricted to ophthalmology science-based multiple-choice questions (MCQs) alone [28]. In general, high SBA performance should be interpreted cautiously as a marker of clinical competence. Singh et al.’s study comparing LLM performance in medical examinations in both SBA and free-text answer formatting found that GPT-4 demonstrated a statistically significant reduction in performance when evaluated using a free-response question format, compared to performance when answering paired SBA questions [29]. Similarly, using midpoint estimates from recent systematic reviews, LLM performance declined by approximately 30% from knowledge-based examinations to clinical competence tasks, with a further 12% decline on safety assessment performance [30]. These findings have led to increasing debate regarding the validity of MCQ-based benchmarking as a proxy for clinical competence.

Educational implications

GPT-5.2 achieved an overall accuracy exceeding 90% in both Instant and Thinking modes, well above the MRCP Part 1 pass mark of approximately 62% [5]. For structured SBA revision where answer options are visible and many questions rely on pattern recognition and selection between plausible distractors, lower-latency modes may provide adequate practical utility. Although Thinking mode showed a small trial-level advantage, the high concordance at the question level suggests that this may not always translate into a meaningful difference for routine question bank use. Although Thinking mode produced fewer errors overall, the question-level analysis of incorrect responses suggests that increased reasoning depth may not uniformly reduce the prevalence of reasoning errors. Instead, it may change the distribution of reasoning errors that occur. This is particularly relevant as medical trainees may assume that longer or more deliberative modes produce more reliable outputs. However, our findings suggest that learners should critically appraise outputs from all operating modes and remain aware that different reasoning modes may be susceptible to different patterns of cognitive bias. Emerging evidence suggests that LLM susceptibility to bias may be influenced by prompting strategy [17,19], although further research is needed to determine whether similar effects occur across different reasoning modes. While LLMs can play an important role in learning and examination preparation, these findings indicate that reasoning errors persist despite use of extended reasoning modes, and so at this stage they must be viewed as adjuncts rather than replacements for traditional study methods and clinical experience.

Limitations

Firstly, our study analyzed only one sample paper of MRCP Part 1 questions; therefore, our findings are not generalizable to the official examination, other examinations, or image-based assessment material. This also limits extrapolation to open-ended clinical reasoning, complex decision-making, and real-world patient care. Furthermore, we did not compare equivalent operating modes in other leading LLMs. We also did not involve human participants to establish baseline performance. It was also not established whether GPT was simply recalling material from a known dataset. However, a previous study found no evidence of memorization of the same question material by GPT-4 using a Levenshtein-distance-based detector [5]. While this finding cannot establish whether GPT-5.2 encountered this material during training, it is unlikely since access to the question content requires email verification and human authentication via CAPTCHA. Response time was not directly measured due to multiple complex confounders, such as networking conditions and device performance. Relative speed inferences are instead based on model design characteristics, such as differences in reasoning structure and token usage. Additionally, only incorrect responses were analyzed qualitatively; thus, it is not possible to determine whether correct answers were produced through clinically sound reasoning, limiting conclusions regarding the educational and clinical safety of model outputs. Although predefined criteria and independent blinded review were used, assigning a single primary error category inherently involved subjective judgment, particularly where responses contained multiple reasoning errors; hence, misclassification bias may be present. Some specialties had very few questions and sparse outcome events, which made fully adjusted models unstable. Similarly, high overall performance suggests a potential ceiling effect, which limits statistical power for paired comparisons and increases the likelihood of a type II error, thus warranting cautious interpretation of our findings. Exact computational reproducibility is limited by the proprietary and evolving nature of LLMs, as no immutable model snapshot, interface build identifier, or internal sampling parameters were available for GPT-5.2.

Suggestions for future research

Future research should move beyond aggregate accuracy and examine how reasoning modes influence the quality, reliability, and educational impact of LLM-generated explanations. Studies should evaluate newer GPT models and other LLMs across multiple examination formats, with datasets sufficiently powered to compare specialties, competencies, and specific bias categories. Future designs should vary reasoning mode, prompt structure, answer format, and the presence of bias-inducing cues while applying a similar predefined error taxonomy. As newer LLMs increasingly offer multiple inference modes, future benchmarking studies should routinely report inference mode alongside model identity. Learner-facing studies should examine revision behavior, misconception formation, and translation of LLM-based revision to examination performance. Bias mitigation strategies should be explored, while also recording latency, token use, cost, and environmental burden to determine when extended reasoning modes are safe and educationally justified.

## Conclusions

Overall, GPT-5.2 Instant and GPT-5.2 Thinking both performed highly on MRCP Part 1 SBA questions. There was little practical separation between scores despite Thinking mode achieving marginally higher odds of a correct trial-level response. The two reasoning modes showed different descriptive distributions of cognitive bias-like errors, although the small number of incorrect responses precludes firm conclusions regarding differences in error patterns. For structured, recall-dominant assessment formats, lower-latency modes may offer comparable practical utility for revision purposes, although learners should be aware of common reasoning errors and critically appraise all LLM-generated responses, irrespective of reasoning depth. Further work should assess bias-mitigation strategies in more discriminating contexts while evaluating explanation quality, safety, and efficiency.

## Figures and Tables

**Figure 4:**
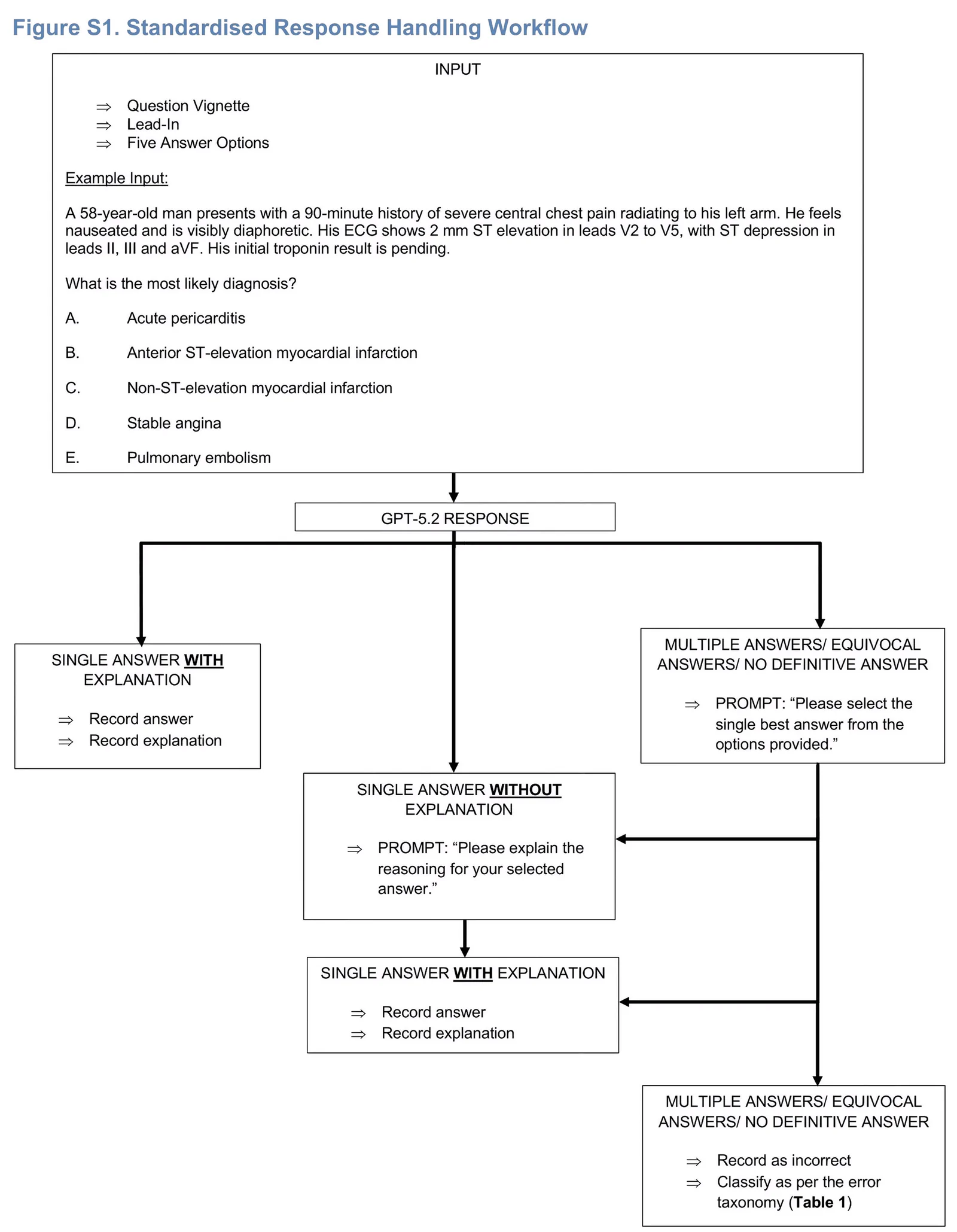
Standardized LLM input and response handling workflow This figure outlines the exact prompt template and workflow when handling GPT-5.2 responses during the data collection process. The acute coronary syndrome question is a hypothetical example used solely to demonstrate the workflow and was not obtained from the official sample paper. Arrows indicate the pathway followed according to the format of the model output. Multiple arrows extending from a box represent mutually exclusive scenarios. “Record answer” refers to inclusion in the quantitative accuracy analysis, while “record explanation” refers to retention of the accompanying explanation (qualitative analysis was limited to incorrect answers only).

## Appendices

Appendix 1 
